# Journal of Ethnobiology and Ethnomedicine – achievements and perspectives

**DOI:** 10.1186/1746-4269-2-10

**Published:** 2006-02-07

**Authors:** Andrea Pieroni

**Affiliations:** 1 Biomedical Research Focus Group/Division of Pharmacy Practice, School of Life Sciences, University of Bradford, Richmond Building (L26c), Richmond Road, Bradford BD7 1DP, UK

## Abstract

Last summer we officially launched the *Journal of Ethnobiology and Ethnomedicine*, published by BioMedCentral, with the aim of establishing a serious, peer-reviewed, open-access online journal that focuses on the multidisciplinary, interdisciplinary, and transdisciplinary fields of ethnobiology and ethnomedicine, drawing on approaches and methods from both the social and biological sciences. The strong start vindicates the widely held belief that the journal responds to a real need within the research community.

The success of the journal has been most gratifying. The steady influx of submissions of high scientific standards illustrates the strong demand for a dynamic, proactive, and *open-minded *scientific journal in these research areas. Our aim has been to dedicate JEE to the "scientific communities" worldwide, particularly those in the developing countries.

## Introduction

Last summer we officially launched the *Journal of Ethnobiology and Ethnomedicine *(JEE) [1], which is published by BioMedCentral, with the aim of establishing a serious, peer-reviewed, open-access online journal that focuses on the *multidisciplinary*, *interdisciplinary*, and *transdisciplinary *fields of ethnobiology and ethnomedicine, drawing on approaches and methods from both the social and biological sciences. The strong start vindicates the widely-held belief that the journal responds to a real need within the research community.

The success of the journal has been most gratifying. The steady influx of submissions of high scientific standards illustrates the strong demand for a dynamic, proactive, and *open-minded *scientific journal in these research areas. Our aim has been to dedicate JEE to the "scientific communities" worldwide, particularly those in the developing countries.

After our launch period ends on February 1st 2006, the journal will be introducing article-processing charges (APC) to cover the costs of publication. These charges will be partly or completely waived for all authors whose institutions are members of BioMedCentral and for scholars in developing countries who are experiencing financial hardship.

## Ethnobiology and ethnomedicine – what they are (not)

A recent editorial in the *Journal of Ethnopharmacology *[2], whose bio-scientific and phytopharmacological rigour is held in great esteem by all of us, lists criteria for immediate rejection of ethnobotanical manuscripts. Included are the following:

*II. No information about the ethnographic background of the study or about the methods used*.

*IV. The ethnopharmacological frame of reference/theory that forms the basis of the study is not spelled out; e.g. no information about how disease diagnosis and practices related to specific plant medical uses were observed and verified*.

*V. No information on the protection of the biodiversity rights of indigenous people or local government*.

In stating these criteria, the editors of the *Journal of Ethnopharmacology*, apart from using a confusing phrasing (ethnographic background and methodologies are not necessarily the same things) have underlined the crucial issue of methodology to be followed during ethnoscientific field studies and how these should serve truly hypothesis-driven research aims and objectives.

On the other hand, a "right of biodiversity to be protected" is very difficult to sustain, at least when expressed in such a form; article 8 (j) of the Convention on Biological Diversity (CDB) [3] states in fact that *each contracting party shall, as far as possible and as appropriate, subject to its national legislation, respect, preserve and maintain knowledge, innovations and practices of indigenous and local communities embodying traditional lifestyles relevant for the conservation and sustainable use of biological diversity, and promote their wider application with the approval and involvement of the holders of such knowledge, innovations and practices, and encourage the equitable sharing of the benefits arising from the utilization of such knowledge, innovations and practices*.

This article of the CDB invites us – much more than to establish further obscure "legal frameworks" – to actively interact with the local communities and all those who retain Traditional Knowledge for finding common strategies and pathways, aimed to improve that common "well-being", which at the end should be the ultimate aim of every scientist.

Further, an open editorial letter recently published in *Economic Botany *[4], a principal reference journal for many of us coming from the ethnobotanical sciences, affirms as a feature of the journal's success the fact that papers submitted for publication now have to go through a reviewing process that lasts only a few weeks, whereas over the previous three years the average length of time it has taken for a manuscript to be published in that journal was not less than eighteen months.

These examples demonstrate how the JEE's broad scope has the potential to attract huge interest within the entire botanical/biological, medical/health and social-scientific communities, and beyond them, but at the same time how difficult it sometimes can be within our (often) secluded academic circuits to keep up with the dynamic changes taking place outside of the scientific community, and especially at the interface between science and society (societies) at large.

I am referring here especially to the crucial issue of the dissemination strategies of the research outputs beyond academia and the very rapidly changing panorama of scientific publishing.

## Challenges in ethnobiology and ethnomedicine

Ethnobiology and ethnomedicine have become crucial for understanding *how people perceive and conceptualise nature, health *(understood here as defined by the WHO [5]) *or other emic concepts of "well-being", and how these issues change over time in response to social, cultural, political or environmental dynamics*.

More importantly, ethnobiology and ethnomedicine now have the chance to change political agendas and to capture the attention of many stakeholders in environmental and health sectors, but only if their sciences, knowledge, practices, experiences, and "know-how" are *widely and freely disseminated*.

In articles so far published in JEE authors have addressed these issues very well in their research on such diverse topics as the role of traditional healers as psychosocial supporters in caring for orphans in Dar-es Salaam city [6], the process and dynamics of traditional sales of wild edible mushrooms in tropical Mexico [7], and fishers' knowledge and seahorse conservation in Brazil [8].

We aim to continue offering JEE as a forum to the wider ethnobiology and ethnomedicine communities throughout the globe. We also aim to turn JEE into a crucial instrument capable to provide analysis and insights for environmentalists, physicians, veterinarians, health-care specialists, development workers, policy makers, and "civil societies", paying special attention to what is going on in the developing countries.

## Article-processing charges

We once again draw your attention to the fact that our publisher, BioMed Central, is introducing article-processing charges (APCs) from February 1st 2006 onwards to recover the cost of publication. APCs are essential for the sustainability of open-access publishing in general and for the success of JEE in particular. Authors should note that open access, made economically viable through APCs, has many advantages over the traditional subscription model with its restrictions on access and the dissemination of knowledge. Authors must be painfully aware that the existence of subscriptions does not exclude page and/or colour charges.

In the open-access model, authors continue to retain copyright ownership of their publications and they do not have to be concerned about the length of their manuscripts or the number of figures and tables as the APC is based on a flat rate. The use of colours does not incur any additional charges, and all articles are archived in perpetuity in the US NIH's PubMed Central and other archives and repositories, with free access over the world -wide web.

Authors affiliated with institutional members of BioMed Central (at present there are over 500 [8]) will continue to enjoy the advantages of membership. In many cases this will include a full waiver of APCs, although in some cases it will be a partial waiver, depending on the membership type. For authors facing hardship, JEE will consider individual requests for waivers. Applications need to be made during the submission process, and a decision on the waiver will normally be made within two working days.

### One more word..

The past six months have been an exciting, fruitful and successful start-up period for JEE. I would like to thank the journal's Editorial Board and all the authors who have shown such great trust in our new initiative.
